# Diagnosis of Disease in Infancy

**Published:** 1947

**Authors:** A. V. Neale

**Affiliations:** Professor of Child Health, University of Bristol


### The Bristol Medico-Chirurgical Journal

*" Scire est nescire, nisi id me*
*Scire alius sciret."*

WINTER, 1947.

### DIAGNOSIS OF DISEASE IN INFANCY

BY

A. V. NEALE, M.D., F.R.C.P.

*Professor of Child Health, University of Bristol*

It is the intention in this paper to consider some of the special problems involved in the early diagnosis of some important diseases in infancy and childhood. Life in the newborn often hangs on a thread, dependent upon the practitioner recognizing the grave danger of delay in concluding the probable sequence of events. It is more important to be confident in the acute emergencies of the newborn than at any other age.

*Aspirational respiratory distress.*—Here a matter of minutes may determine success or failure. Even ten minutes of respiratory anoxaemia may lead to irreparable damage to important nerve cells in the basal ganglia.

*Cerebral oedema* may arise, especially under conditions of protracted labour or as a consequence of Caesarean section. Neonatal " asphyxia pallida " or a firm fontanelle may suggest the diagnosis. A minor convulsion or twitching might occur. Major convulsions, deeper flaccidity, and fundal haemorrhage indicate intracranial haemorrhage. The cerebral oedema is usually temporary (24-48 hours) and causes some delay and limitation of the normal cry, sucking movements and muscle tone ; and especially respiratory variation with possibly alarming periods of bradypnoea. Treatment : Lobeline may be given : with a minimum of handling, adequate warmth, drops of glucose water by mouth, and a " coramine cocktail " (coramine or nikethamide *m* v ; brandy *m* xx,

O

warm water ʒii) repeated each two or three hours. Twenty c.cm. of 10 per cent. saline per rectum may reduce intracranial pressure and cerebral oedema.

*Acute pulmonary oedema* may be recognized by the cyanosis, bubbling respiratory sounds, obstructive type of breathing and very rapid heart rate. Atropine sulphate gr. 1/250, repeated after an hour or so, may check the condition.

*Hypoglycaemia* is a possible factor in neonatal collapse. The blood sugar of a newborn baby fluctuates rather widely because the glycogen-glucose balance is relatively unstable. This rarely causes serious signs, except in a newborn whose mother is a diabetic: collapse with sweating, flushing, twitching and possible convulsive movements should suggest the diagnosis. Glucose should be given, if necessary by stomach tube, and small doses of adrenalin (*m* i–ii) by injection. Diabetic mothers tend to produce overweight babies.

*Haemorrhagic disease.*—With the discovery of vitamin K and its relationship to prothrombin synthesis paediatricians were hopeful that neonatal haemorrhagic disease would disappear. Unfortunately preventable deaths still occur. Remember that a baby, especially if premature, tends to fail in its plasma prothrombin level at about the third to eighth day of life, with a consequent possibility of spontaneous and profuse mucosal bleeding, most commonly gastro-intestinal (haematemesis or melaena neonatorum). The loss of one or two ounces of blood may be fatal. In the established disease vitamin K intramuscularly is essential; blood transfusion or intramuscular blood (this latter alone is not always efficacious) should not be delayed if clinical recovery is not quick and certain.

*Sepsis.*—Constant vigilance is needed against infection, especially skin and respiratory infections. A high percentage of neonatal sepsis is due to staphylococci. Therefore no one with known infection should handle babies. A small boil in a baby has grave potentialities: pyaemia, infantile osteomyelitis, septic pericarditis, etc. The virus of the "common cold," a transient nuisance to an adult, is to a baby of profound gravity. Neonatal pneumonia is practically always a "contact" disease which may spread round a nursery at great speed and needs immediate recognition and treatment. Pyrexia, loose stools, occasional cough, quickened breathing, active alae nasi, and variable cyanosis are enough for clinical diagnosis. Quite quickly, signs appear in the lungs with bronchiolitic breathing and lower intercostal retraction. The combination of acute bacterial toxaemia and respiratory anoxaemia may lead to sudden collapse. Penicillin is usually a life-saving measure if given early: an oxygen tent, providing oxygen at about 40 per cent. concentration, is most valuable.

*Pyloric stenosis.*—Early diagnosis in pyloric stenosis increases the chances of recovery. There is no reason why with early diagnosis and continued breast feeding, every baby with pyloric stenosis should not recover. The diagnosis is not difficult if the possibility is borne in mind with every vomiting baby.

*Paroxysmal tachycardia* occurs in very early infancy. It may be due to simple auricular tachycardia, auricular flutter or auricular fibrillation, which are diagnosable by electrocardiogram.

Many cases gradually eliminate the tendency and the attacks become less frequent and alarming. Prevention of air in the stomach, small frequent feeds, and a minimum of handling, are essential.

| | |
|---|---|
| ℞ Digoxin | 0.25 mg. |
| Coramine | 0.5 mil. |
| Sp. V. Gall. | 1.0 mil. |
| Aq. ad | 8 mil. |

Sig. gutt x o.h.

*Gastro-enteritis* has a higher danger level in babies round six to nine months of age than in the younger babies. Nevertheless, throughout the first two years of life this disease is always serious, since its well-known tendency to relapse and death from dehydration. We are, in these cases, often faced with a complex biochemical derangement. However, again early diagnosis is the keynote. The primary cause of acute infantile gastro-enteritis is not yet determined, but we do know that in a very high proportion of cases there is an associated upper respiratory infection : otitis media may be present. In some cases, the gastro-enteritis is associated with acute pyelitis. The presence of streaks of blood in the stools may suggest direct infection of the intestinal mucosa with streptococci. In some small percentage of cases the diarrhoea and vomiting are due to primary alimentary infection. Treatment of the acute gastro-enteritis of infancy includes particular attention to body fluids and metabolic factors as well as every means to combat causative or associated infection. Hospital treatment is often essential. A baby with an upper respiratory infection is very liable to acute diarrhoea and vomiting, but it is somewhat unusual for anything more than mild diarrhoea to occur in a baby who has acute pneumococcal bronchopneumonia. An abscess, or an empyema, may be quite benign in its general effects. One might almost regard pus (when it can be adequately evacuated) in a "laudable" sense under these circumstances.

*Acute bronchiolitis* is one of the most dangerous forms of lower respiratory disease in infancy or childhood. The very narrow bronchioles may be quickly blocked by mucopurulent exudate added to congested mucosa ; and finally secondary spasm of the relatively thick musculature will completely occlude the lumen. Inspection of the chest reveals the inspiratory and expiratory difficulty, and auscultation reveals signs of focal lung collapse, etc. Cyanosis and great respiratory distress may be present. An oxygen tent is usually essential in handling such a condition. An urgent attack on the infection with penicillin and sulphonamide is necessary.

*Acute scurvy* remains a dreadful disease in babies and every doctor should beware of the situation when the mother reports that her baby (usually aged about nine months) is irritable, unhappy and resents the legs and thighs being handled or moved. This should suggest early scurvy. An inspection of the gums (if teeth are present) will show deep purple swelling : there is danger of periosteal haemorrhage and epiphyseal separation. Two cases seen recently of unrecognized scurvy succumbed to visceral and intra-cranial haemorrhage. Some oral infections may be aggravated by deficiency in riboflavine and nicotinic acid. It is as well to remember that nutritional disease in early childhood may be related to unsuspected steatorrhoea.

*Appendicitis* at any age may be a clinical difficulty, especially under one year. The appendix vermiformis is not infrequently placed in " atypical " positions. There is a special tendency to a rapid perforative appendicitis and in fact most cases are recognized only at the stage of generalizing peritonitis. Acute general peritonitis, with a rigid, distended, diffusely tender abdomen, and possibly a recent history of acute diarrhoea, followed by constipation, should suggest the diagnosis in the baby.

*Acute pneumococcal peritonitis* may occur in young children (more often girls). The clinical story and picture may resemble that of appendicitis very closely. In the pneumococcal case there is usually a temperature reaching 103°–104°, flushed face with peri-oral pallor, quickened breathing and hot, dry skin. The abdomen is generally distended and tender, but usually not so rigid as in the infection resulting from acute appendix disease. If one is confident of the diagnosis, surgery is avoided and reliance can be placed upon penicillin. Toxic peritonism in acute basal pleuro-pneumonia should not be confused with true intra-peritoneal disease.

*Melaena* in infancy is always very alarming. In the early days of life the appearance of dark blood usually indicates a state of prothrombin deficiency : rarely, bleeding may occur from an acute duodenal ulcer. Also, rarely, the cause of the bleeding may be a congenital deficiency of fibrinogen. Congenital colonic or rectal polypus may be responsible : treatment is surgical. In any case of obscure and recurrent melaena at this age, exploration may be necessary. The absence of melaena does not exclude intussusception. Vomiting, combined with the observed anguish in the baby's face during an attack of intussusceptic colic, leaves little doubt about the problem. In older children, acute dysenteric ulcerative entero-colitis may be alarming when viewed from the point of view of the appearance of the frequent and heavily blood-stained motions ; however, therapy, including alimentary sulphonamides, is often quickly effective. In the more refractory state of juvenile ulcerative colitis, the disease process more often involves the descending and sigmoid areas of the colon and there is a troublesome possibility of profuse bleeding from vessels in the inferior mesenteric vascular distribution. An uncommon disease—Crohn's regional ileitis (which histologically resembles tuberculous disease but in which no tubercle bacilli have been found)—may indicate its presence by bouts of melaena.

